# Optimizing COVID-19 testing strategies on college campuses: Evaluation of the health and economic costs

**DOI:** 10.1371/journal.pcbi.1011715

**Published:** 2023-12-22

**Authors:** Kaitlyn E. Johnson, Remy Pasco, Spencer Woody, Michael Lachmann, Maureen Johnson-Leon, Darlene Bhavnani, Jessica Klima, A. David Paltiel, Spencer J. Fox, Lauren Ancel Meyers

**Affiliations:** 1 Department of Integrative Biology, The University of Texas at Austin, Austin, Texas, United States of America; 2 The Pandemic Prevention Institute, The Rockefeller Foundation, New York, New York, United States of America; 3 Santa Fe Institute, Santa Fe, New Mexico, United States of America; 4 Department of Population Health, Dell Medical School, The University of Texas at Austin, Austin, Texas, United States of America; 5 Office of the Vice President for Research, The University of Texas at Austin, Austin, Texas, United States of America; 6 Public Health Modeling Unit, Yale School of Public Health, New Haven, Connecticut, United States of America; 7 Department of Epidemiology & Biostatistics, The University of Georgia, Athens, Georgia, United States of America; University of Washington, UNITED STATES

## Abstract

Colleges and universities in the US struggled to provide safe in-person education throughout the COVID-19 pandemic. Testing coupled with isolation is a nimble intervention strategy that can be tailored to mitigate the changing health and economic risks associated with SARS-CoV-2. We developed a decision-support tool to aid in the design of university-based screening strategies using a mathematical model of SARS-CoV-2 transmission. Applying this framework to a large public university reopening in the fall of 2021 with a 60% student vaccination rate, we find that the optimal strategy, in terms of health and economic costs, is twice weekly antigen testing of all students. This strategy provides a 95% guarantee that, throughout the fall semester, case counts would not exceed twice the CDC’s original high transmission threshold of 100 cases per 100k persons over 7 days. As the virus and our medical armament continue to evolve, testing will remain a flexible tool for managing risks and keeping campuses open. We have implemented this model as an online tool to facilitate the design of testing strategies that adjust for COVID-19 conditions as well as campus-specific populations, resources, and priorities.

## Author summary

As a part of the COVID-19 response team at a large public university in the US, we developed an online analytic platform to aid administrators in weighing the costs of massive testing programs against the potential risks of letting COVID-19 spread unchecked. We apply the approach to derive a cost effective screening policy for a large public university in the fall of 2021. By then, SARS-CoV-2 tests were relatively inexpensive and widely available, yet many universities were legally prevented from requiring testing, the Delta variant was surging, and vaccine coverage among college students was limited. We demonstrate the versatility of the approach for tailoring university mitigation strategies depending on the nature of the pathogen threat, the composition of the campus population, and the resources and priorities of the institution.

## Introduction

During the first two years of the COVID-19 pandemic, colleges and universities throughout the US struggled to provide in-person education while mitigating the health and economic risks of COVID-19. Decisions regarding campus COVID-19 policy were often difficult because of complex logistical, economic and societal impacts. Large outbreaks on campus not only jeopardized the health and safety of students, employees, and the surrounding communities, but also exacted significant economic and logistical costs to the universities and nearby healthcare systems [1–6]. Full campus closures, an extreme but common early response, also posed significant financial, educational, and reputational costs to the university. Many universities therefore sought more moderate policies for safely reopening and maintaining in-person educational, recreational, and residential activities.

By the summer of 2021, the arrival of vaccines [7] and the increased availability of rapid diagnostic tests allowed universities to restore many of the key elements of the residential campus experience. Early studies demonstrated that frequent testing of asymptomatic people to reduce transmission––screening testing––could be a powerful and versatile tool for mitigating risks [8–10]. However, the approach was not widely or consistently applied to safeguard university campuses [4,6,11]. A few universities launched large COVID-19 screening programs and reopened fully in the fall of 2020 based on analysis showing that asymptomatic screening testing once or twice per week would be sufficient to both track and mitigate COVID-19 risks, even in the absence of a vaccine [8,12]. However, many asymptomatic testing programs were designed as small-scale surveillance systems to track prevalence rather than large-scale mitigation programs to prevent transmission [13,14]. Some universities reduced costs through pooled testing, in which large numbers of samples were combined for batch testing followed by individual testing of positive batches only [15]. Asymptomatic testing services were often separated from the symptom-based testing programs available on most campuses to support rapid diagnosis, provision of healthcare, and initiation of isolation, contact tracing and quarantine efforts [6,7,16]. Early in the pandemic, prior to the wide availability of rapid antigen tests, many campuses had difficulty securing sufficient laboratory resources to process large numbers of Polymerase Chain Reaction (PCR) tests. During surges, many university labs were overwhelmed, resulting in turnaround times ranging from a few days up to over a week [17,18]. Universities also struggled to keep up with contact tracing and the demand for isolation rooms. Regardless of testing strategy or resource availability, many US universities opted to close campuses and resume online instruction during major surges [19,20]. The variable success and limited implementation of COVID-19 screening programs throughout the first two years of the pandemic highlights the importance of designing policies that balance institutional costs and public health benefits, while adapting to continually changing resources and risks.

Although large-scale screening testing offers a viable but underutilized strategy for reducing transmission in a high contact setting like a university campus [8,21,22], there remains a lack of clarity regarding the types of students and staff that should be screened and the frequency of screening, as both the severity of the virus and immunity from prior infection and vaccination continue to evolve. Moreover, the costs of screening testing include not only direct investment in testing resources, but also indirect impacts on individuals and organizations stemming from increases in the detection rate for asymptomatic and mild infections. Mathematical models that capture these complex tradeoffs can help to guide universities in tuning SARS-CoV-2 screening policies to balance costs and benefits as epidemiological risks evolve.

Throughout the COVID-19 pandemic, infectious disease modelers worked closely with university administrators to provide situational awareness and decision-support tools to guide campus policy [2,12,23–27]. Data-driven models of SARS-CoV-2 transmission on campuses were used to provide projections that estimated the impact of various policies on disease spread and burden across a range of scenarios. Such models helped to design screening testing policies that maximized in person-days [2], depending on the campus vaccination rate [2,12] and risk thresholds set by the university [25]. Other studies have demonstrated the cost-effectiveness of screening testing for the community at large [9,10,28].

Here, we introduce a more detailed framework for designing campus SARS-CoV-2 policies that can guide universities depending on their risk tolerance, resource availability, the composition and immunity of their student and staff populations, the severity and prevalence of circulating variants, and the availability and efficacy of vaccines. The underlying transmission dynamic model considers a well-mixed, partially-vaccinated student population following a specified testing policy. We developed this approach to support planning efforts at the University of Texas at Austin (UT Austin), one of the largest public universities in the US, during the summer of 2021. As a case study, we apply it to design a cost-effective SARS-CoV-2 screening policy to prevent campus closures in a partially-vaccinated community of 50,000 students during the emergence of a novel variant (Delta). At the time of the original analysis, we had estimated that about 60% of the UT Austin student body had been fully vaccinated, based on county-level age-specific vaccination data [29]. Given the uncertainty in that estimate and the opportunities to increase coverage prior to the start of the semester, we considered a range of vaccination rates. By integrating both the health and economic costs of COVID-19, testing, and campus closures into a transmission dynamic model, the framework provides more comprehensive information for decision makers. It is available as an online tool [30] to support US universities in tailoring infectious disease screening programs as novel SARS-CoV-2 variants and other pathogens threaten the health and safety of campus communities.

## Results

**Fig 1:**
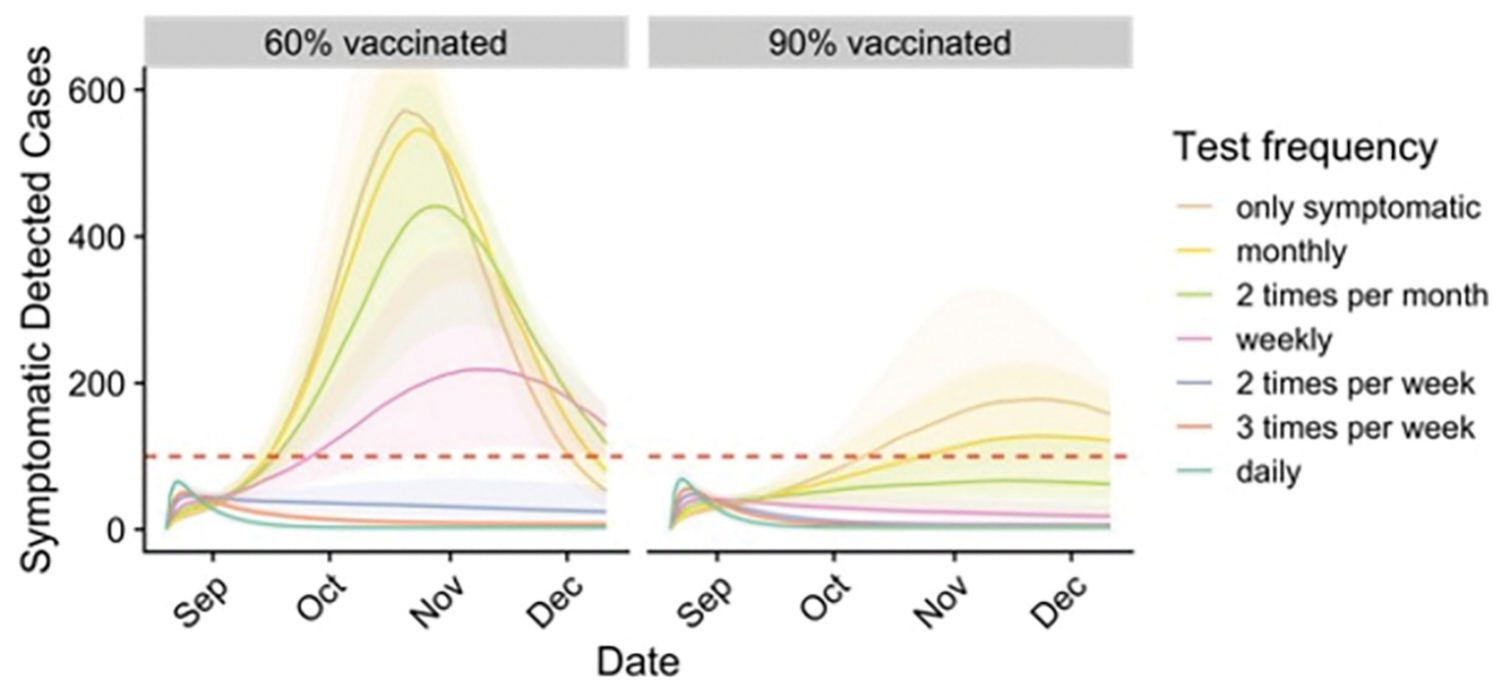
Projected COVID-19 cases among students under different levels of screening testing, assuming 60% (left) or 90% (right) of students are fully vaccinated. Graphs project the seven-day total of detected symptomatic cases in a campus of 50,000 students. Colors indicate testing frequency assuming 75% compliance. Shading indicates 90% prediction intervals. Dashed horizontal lines represent the assumed campus closure threshold (twice the CDC’s high COVID-19 transmission threshold). The optimal testing frequency for each vaccination rate is the one which ensures the upper bound of the 90% prediction interval remains below the campus closure threshold. At a 60% or 90% vaccination rate, this corresponds to testing all students twice or once per week, respectively.

In the summer of 2021, we derived an optimal screening testing strategy for UT Austin, an urban public university with 50,000 students, for the upcoming fall semester. Given the uncertainty in vaccination coverage at the time, we considered a range of vaccination rates (Fig 1 and Table 1 and Figs B, C, D, E, and F in S1 Text file). If 60% of students arrive vaccinated, we project that cases could far surpass the US Centers for Disease Control and Prevention (CDC) threshold for high COVID-19 transmission, potentially triggering a campus closure. With only symptom-based testing (i.e., testing only symptomatic patients seeking care), we estimate that symptomatic case counts would peak between 460 and 710 (median: 575) per week in mid-October. If 75% of all students test two times per week the expected peak reduces to 40–60 (median: 50), with a 95% guarantee of remaining below the closure threshold of 100 cases per 100k in 7 days (twice the CDC’s threshold for high COVID-19 transmission). This optimal strategy would require approximately 75,000 tests per week. If 90% of students are vaccinated, however, weekly testing would be sufficient to prevent a campus closure.

**Table 1:** Recommended testing levels under three different policy options, across a range of vaccination rates. Testing recommendations are based on the minimum amount of testing needed to provide 95% certainty that symptomatic infections will not exceed the campus closure threshold across a range of vaccination rates. For each optimal policy, the number of tests per person per week and total number of tests are reported.

Population of students tested	Percent of students fully vaccinated				
	50%	60%	70%	80%	90%
**Number of tests per person per week**					
**All**	3	2	2	2	1
**Unvaccinated at twice the rate of vaccinated**	3 (unvacc) 1.5 (vacc)	3 (unvacc) 1.5 (vacc)	2 (unvacc) 1 (vacc)	2 (unvacc) 1 (vacc)	2 (unvacc) 1 (vacc)
**Only unvaccinated**	7	7	7	7	Not possible*
**Total number of tests per week**					
**All**	112,500	75,000	75,000	75,000	37,500
**Unvaccinated at twice the rate of vaccinated**	84,375	78,750	48,750	45,000	41,250
**Only unvaccinated**	131,250	105,000	78,750	52,500	Not possible*

* Due to the very small size of the population being tested, if vaccination rates are very high and testing only is occurring in the unvaccinated, it is not possible to ensure that the campus closure threshold won’t be exceeded

**Fig 2:**
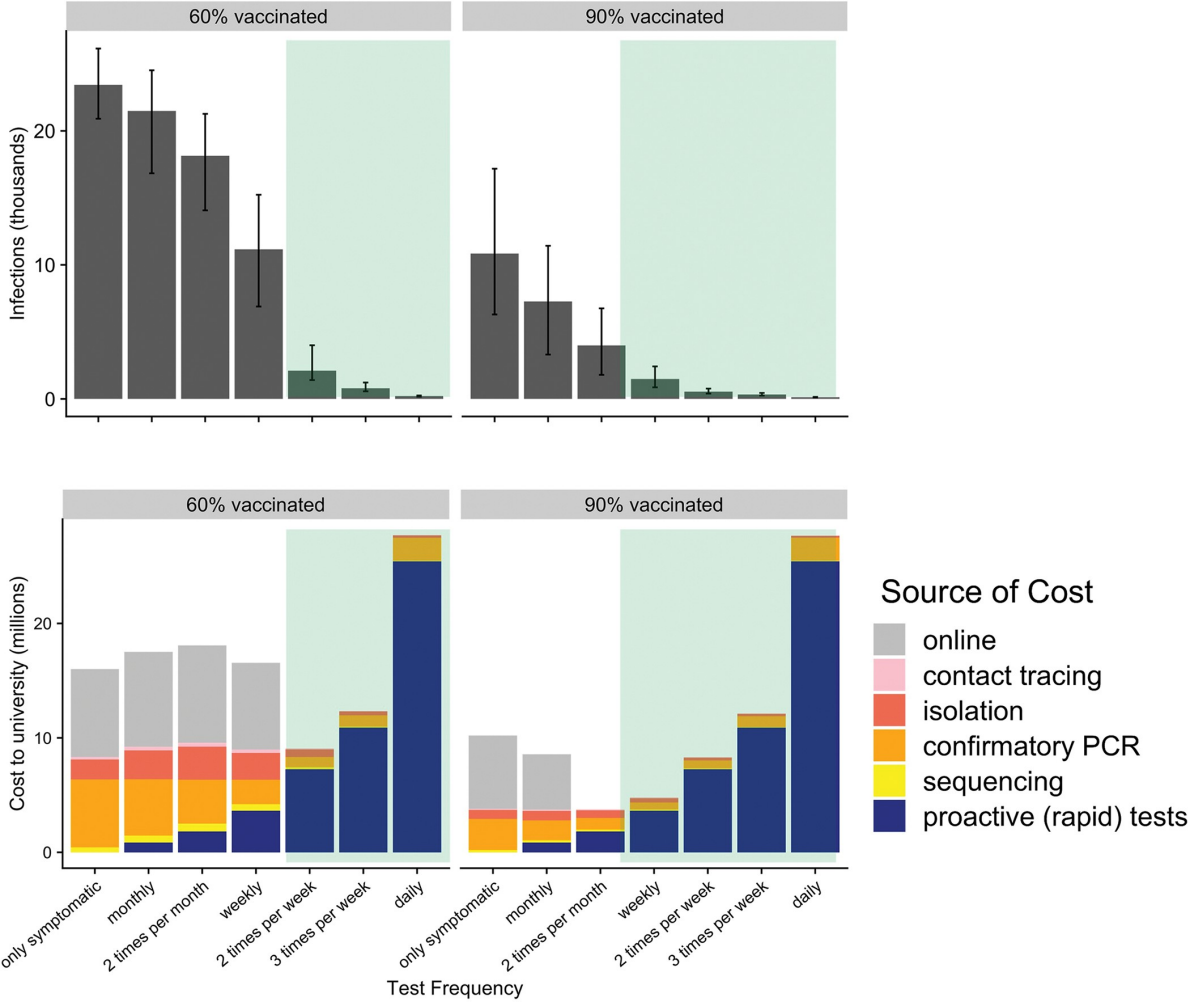
Projected health and economic costs over one semester under different levels of screening testing, assuming 60% (left) or 90% (right) of students are fully vaccinated. Upper graphs indicate the median and 90% predictive interval of projected cumulative infections under the corresponding testing policy (x-axis). Lower graphs indicate the projected costs of each testing policy, broken down by the source (colors), with the gray indicating the median expected cost of a campus closure (i.e., transitioning from in-person to online instruction). The green shading indicates testing frequencies that have a 95% guarantee of preventing reported symptomatic cases from exceeding the university’s closure threshold, as illustrated in Fig 1.

The optimal testing frequency depends on both vaccine coverage and whether vaccinated students are exempt from testing (Table 1 and Fig D in S1 Text file). At 90% vaccine coverage, testing only the unvaccinated would be insufficient, given our assumption that vaccines reduce susceptibility to infection by only 47% [31,32]. Across vaccination rates, exempting vaccinated students from testing requires frequent (daily) testing of unvaccinated students to prevent a surge, costing more testing resources than if all students regardless of vaccination status were tested. Testing vaccinated students at half the rate as unvaccinated students remains a viable option, as total testing resources are, at most vaccination rates, lower than if all students were tested (Table 2). At 70% vaccine coverage, testing the unvaccinated twice per week and the vaccinated weekly requires 48,750 tests per week compared to 75,000 if vaccinated and unvaccinated test at equal rates. Across testing frequencies, the costs and infections associated with either prevention (screening tests) or outbreak response (contact-tracing, isolation, sequencing, confirmatory PCR) are expected to be significantly higher under 60% vaccine coverage than 90% vaccine coverage (Figs 2 and Fig C in S1 Text file).

**Table 2:** Estimated levels of screening testing, tests per week, and health and economic costs associated with the optimal testing policy at each vaccination rate. The total cost includes the direct costs of screening testing and the cost of pandemic response-related expenses (i.e., confirmatory testing, isolation, contact tracing, sequencing).

	Percent of students fully vaccinated				
	50%	60%	70%	80%	90%
**Minimum frequency (in all students)**	3 times per week	2 times per week	2 times per week	2 times per week	weekly
**Total proactive tests per week**	112,500	75,000	75,000	75,000	37,500
**Total cost to university ($) if testing implemented**	$12.5 million	$9.1 million	$8.7 million	$8.4 million	$4.7 million
**Cost of testing per student**	$218	$145	$145	$145	$73
Cost of testing per infection averted*	$442	$342	$366	$463	$379
**Number of infections expected if only symptomatic testing is offered**	25,600	23,600	20,400	17,000	10,800

*The number of infections averted is estimated by comparing the projected incidence with and without testing, assuming no differences in behavior under the two scenarios. In reality, the absence of testing might lead to campus closures and other mitigation measures that reduce infections.

At 60% vaccine coverage, screening testing all students twice per week is sufficient to avoid exceeding the campus closure threshold at an estimated cost of around $9.1 million (Table 2). At 90% vaccine coverage, screening testing of all students weekly is sufficient to avoid closure at a cost of $4.7 million (Table 2). We note that it costs nearly twice as much ($9.1 million vs $4.7 million, Fig 2 and Table 2) to avoid campus closure at 60% vaccine coverage than at 90% vaccine coverage.

At both the 60% and 90% vaccination rates, the optimal frequency of testing would be cost saving or cost nearly the same as the resources required for outbreak response. At insufficient testing levels, the costs of outbreak response (i.e. contact tracing, isolation, confirmatory PCR, and sequencing) are expected to be at least as high as the costs of screening testing. If we assume that the university will move to online instruction once reported cases surpass the campus closure threshold, then screening testing at the recommended levels is always cost-saving. Optimal screening can significantly lower infection rates. At 60% vaccination, the optimal twice-weekly screening is expected to result in only 2,280 infections over the course of the semester (113 days), whereas symptomatic-only testing results in an expected 23,700 infections (Table 2).

## Discussion

SARS-CoV-2 screening programs can help to suppress transmission on US university campuses and be cost saving. Substantial investments in testing materials and personnel may ultimately avert even higher costs associated with outbreak response activities and campus closures. At a large university in the fall of 2021, we find that the costs of an effective screening program would have been relatively modest, between $73 and $218 per student for the entire semester. As we confront newly emerging variants of COVID-19 and novel pathogens, screening testing may serve as a politically tractable and cost-effective mitigation strategy in college communities with low levels of population immunity.

Our projections suggest that, even at high vaccination rates, testing only unvaccinated students is insufficient to avoid a surge. The two other policies considered––testing all students equally and testing unvaccinated students at twice the rate of vaccinated students––are expected to be more effective without exacting additional costs. We recommend that universities make use of the online tool [30] and framework presented here to develop screening testing policies that align with campus priorities while preventing the high costs of outbreak response.

During the first two years of the SARS-CoV-2 pandemic, large-scale screening was a nimble mitigation tool for universities facing novel variants, changing levels of immunity, and shifting attitudes towards face masks and intrusive social distancing measures. Our study provides a tool tuning screening targets and frequency to match the changing risks and achieve university goals. As levels of immunity and variant severity evolve, universities can scale accordingly to safeguard in-person activities. This approach can be extended to provide ongoing policy guidance based on real-time case reports and other surveillance data.

Although the recommended screening policies were designed specifically to help UT Austin manage the Delta variant wave, our method and qualitative insights are broadly applicable to outbreak mitigation on US college and university campuses. Our online tool allows users to adjust both university-specific inputs, including the community composition and the costs associated with testing and other interventions, and disease-specific inputs, including transmission characteristics and levels of population immunity [30]. The risks of COVID-19, the efficacy of interventions, and costs associated with both may depend on university policies, student behavior, vaccine uptake, and the emergence of variants with different levels of transmission, immune evasiveness, and severity.

We note that our projections assume a high and constant transmission rate throughout the simulation period, and thus do not account for increases in face mask usage and other cautionary behaviors as perceived risks increase during surges. The model assumes that 75% of students would participate in screening, which may require aggressive outreach and also depend on changing risks. Our assumption that individuals fully isolate following a positive test may be unrealistic [33] or require additional isolation rooms, paid sick-leave, and removal of academic penalties for missed classes. We do not explicitly model the waning of infection-acquired or vaccine-acquired immunity; nor do we consider the health or economic costs of severe disease, long COVID, or mortality, variation in risks across students, faculty, and staff, or the cost to the individual of missing class during their isolation period following a positive test.

Prior studies have demonstrated that frequent SARS-CoV-2 screening can reduce transmission [8,22,23,25] and be cost-effective [9,10,28]. A similar decision-support tool helps universities optimize testing while keeping cumulative cases below 5% of the population [25]. However, our approach is the first to explicitly consider diverse health and economic costs associated with screening, isolation, and campus closures. The CDC [34] and American College Health Association (ACHA) [7] have continually released guidance to help universities navigate the changing risks of COVID-19. As the virus continues to evolve and new pandemic threats emerge, our tool will allow campus decision makers to tailor such guidance to the specifics of their institution.

## Materials & methods

### Ethics statement

This study was determined to be “not human subjects research” by the University of Texas at Austin Institutional Review Board.

### Transmission model

We developed a compartmental Ordinary Differential Equation (ODE) model of SARS-CoV-2 transmission that incorporates vaccination and isolation. A full description of the model structure and parameters are provided in Section A in S1 Text file. In short, the model divides the population into four subgroups based on vaccination status and isolation status. While individuals remain in the same vaccination state throughout the semester (duration of the simulation), they can transition between the active and isolated states as a result of receiving a positive test result or developing symptoms. Individuals transition between disease states (Susceptible, Exposed, Asymptomatic-Infectious, Symptomatic-Infectious, Recovered) throughout the course of their infection, with a fraction of infectious individuals never developing symptoms. We assume that over the course of the semester, individuals who have recovered from infection either shortly before the start of the semester (modeled as the initially recovered proportion) or who have recovered from infection during the semester, have complete protective immunity from infection. We also assume a constant vaccine effectiveness against infection, symptoms, and onward transmission separately, throughout the duration of the semester.

In our UT Austin case study, we modeled COVID-19’s characteristics during the summer of 2021, immediately after the Delta variant rose to dominance. We assumed that vaccines reduce the risks of infection by 47% [95% CI: 37–50%] [31,32], reduce the likelihood of developing symptoms by 64% [95% CI: 63–73%] [32], and reduce transmission to others by 20% [35]. We assume a reproduction number (*R*_0_) of 5 without interventions, and that the transmission rate in the active population remains constant throughout the semester. We assume that 75% of students comply with testing policies, and that the majority (92.5%) of students who test positive isolate for seven days. A full list of initial conditions and parameter values can be found in Tables A and B in S1 Text file. The modeling framework allows for different testing rates in the vaccinated and unvaccinated populations. Our case study assumes that the rates are identical; results for different rates are provided in Table 2 and Section C in S1 Text file. We did not explicitly model the effect of quarantining close contacts on reducing transmission, nor do we incorporate directly behavioral changes that may result as cases rise. Finally, we assumed that 25% of symptomatic individuals infected with SARS-COV-2 would seek testing. We tracked the rolling seven-day total detected symptomatic cases, where cases are detected through both screening (antigen) testing and symptom-based care seeking.

### Economic model

To estimate the total cost of each scenario, we consider both the costs of testing and the costs associated with SARS-CoV-2 outbreaks on campus. For our case study, the cost parameter values were set by expert university administrators (e.g., sequencing costs were estimated in consultation with sequencing labs; isolation facility costs were estimated in consultation with housing personnel). All economic factors considered and their associated costs can be found in Table C in S1 Text file. To estimate the costs of testing, we considered both testing supplies and the personnel needed to administer tests, collect data, and process results. All screening testing was assumed to be performed via antigen testing, at a significantly reduced cost than PCR tests. We assumed that all positive screening tests were followed by a PCR confirmatory test. Symptom-based care seekers received only a PCR test. PCR confirmation was then followed by contact tracing, molecular sequencing of the test specimen, and a recommended seven-day isolation period. At the time of the case study, contact tracing was conducted and close contacts of positive cases were encouraged to get tested. We assumed that 20% of positive cases require a campus-provided isolation room, based on the proportion of UT Austin students living in shared on-campus housing. Finally, we considered the costs of campus closures triggered by large surges in cases. Based on conversations with university leadership, we assume that on-line instruction incurs additional costs of $100,000 per day. We do not explicitly consider educational losses from missed classes, administrative costs of coordinating COVID-19 responses, or the healthcare costs associated with student illness. For each scenario, we use the results from the transmission model (e.g., number of tests performed, number of positive tests, days above the campus closure threshold) to estimate the costs associated with each testing policy.

### Campus closure thresholds

To model different campus risk tolerances, we assume that administrators would trigger a transition to hybrid or online instruction (campus closure) when reported case counts surpass one of the following public health thresholds [36].

- High risk: 100 detected symptomatic cases per 100,000 people in a seven-day period, corresponding to the original CDC red (high) alert level.
- Higher risk: 150 detected symptomatic cases per 100,000 people in a seven-day period, corresponding to the 1.5 times the original CDC red (high) alert level.
- Very high risk: 200 symptomatic detected cases per 100,000 people in a seven-day period, corresponding to double the original CDC red (high) alert level.

Our case study assumes that the university would close when the seven-day new symptomatic case count exceeded the very high risk threshold (corresponding to 100 cases in the past 70 days in a population of 50,000). In our online tool, we provide even higher thresholds to support universities in mitigating highly transmissible variants with lower severity, like Omicron [37].

### Identification of optimal testing levels

We considered campus vaccination rates ranging from 50% to 90% in 10% increments and screening testing rates ranging from symptomatic testing only (no screening) to daily screening tests. For a given level of vaccination, we identified the minimum frequency of screening testing required to ensure that the university does not exceed its closure threshold, with a 95% guarantee. For each candidate screening policy, we ran 100 deterministic simulations for the duration of the semester (113 days in the UT case study), each with parameters randomly selected from their specified distributions (Table B in S1 Text file), and identified the lowest frequency screening policy in which 95% of simulations remain under the closure threshold. We identified the optimal policy conditioned on the vaccination rate; across vaccination rates, the costs associated with either screening testing or outbreak response generally increase as the vaccination rate decreases.

### Sensitivity analyses

We conducted a sensitivity analysis with respect to vaccine effectiveness against infection (ranging from 40%-90%, base case at 47%), vaccine effectiveness against onwards transmission if infected (ranging from 50% to 0% effective, base case at 20%), and the testing policy (only unvaccinated, unvaccinated at double the rate of vaccinated, and all students equally). The results are provided in Section C and D, Table D and Fig E in S1 Text file.

## Supporting information

S1 Text Supplement to Optimizing COVID-19 testing strategies on college campuses: evaluation of the health and economic costs. Section A. COVID-19 Transmission model with vaccination and testing. Fig A. Compartmental model of COVID-19 transmission incorporating testing and vaccination. Table A. Initial conditions. Table B. Transmission model parameters. Table C. Cost parameters. Section B. Results for vaccination coverage ranging from 50% to 90% with all students tested. Fig B. Projected COVID-19 cases among students under different levels of screening testing, assuming 50%, 60%, 70%, 80%, and 90% vaccination coverage amongst students. Fig C. Projected health and economic costs through December 16, 2021 under different levels of screening testing, assuming 50%, 60%, 70%, 80% or 90% of students are fully vaccinated. Section C. Results for vaccine coverage ranging from 50% to 90% and different populations tested. Fig D. Projected COVID-19 cases among students under different levels of screening testing, assuming 50%, 60%, 70%, 80%, and 90% vaccination coverage and in testing policies for all students, testing vaccinated at half the rate, and testing in the unvaccinated only. Table D. Estimated level of screening testing to provide 95% guarantee that symptomatic cases will remain below the *very high risk threshold* if testing at half the rate in vaccinated vs unvaccinated. Table E. Estimated level of screening testing to provide 95% guarantee that symptomatic cases will remain below the *very high risk threshold* if only the unvaccinated are tested. Section D. Sensitivity analysis: vaccine efficacy against infection and transmission. Fig E. Projected COVID-19 cases among students as a function of the vaccine efficacy against infection and symptomatic disease assuming 50%, 60%, 70% or 80% of students are fully vaccinated. Fig F. Projected COVID-19 cases among students as a function of the vaccine efficacy against transmission assuming 50%, 60%, 70% or 80% of students are fully vaccinated. Section E. Modifications to framework/Rshiny app for future variants. Table F. Rshiny app default settings and suggested adjustments for Omicron.
(PDF)
